# Mr. Weaver, on Cancer and Fungus Hæmatodes

**Published:** 1807-12

**Authors:** J. W. Weaver

**Affiliations:** Surgeon. Walsall


### *To the Editors of the Medical and Physical Journal.*

GENTLEMEN,

FROM practical experience as well as minute investigation of the affinity peculiar to the various diseases incident to the human body, I am not disposed to coincide with Mr. Shearly in the opinion relative to the analogy subsisting between Cancer and the "Fungus Hæmatodes;" the former, no doubt, is an extreme, or species of scrophula, generally constitutional, et sine causa externa evidenta, tho' it is possible the disease might be brought into action by, or or in consequence of a blow, so as to affect the glandular system, yet the instances are few, and not sufficient to substantiate the conjecture. The variolous inoculation is well known to produce the effect, by rousing the latent seeds of scrophula into action, contrary to the opinion of many eminent professors, who maintain, that it is not capable of producing any other disease, than that of its own nature or kind. As it is necessary to be explicit, in order to be understood, must observe, that I write to convey the idea, that, generally speaking, I cannot conceive that cancer is ever produced by a blow, or any local injury, without the system being previously affected by scrofula, which leads me to observe, that the fungus hæmatodes is essentially different, and produced from different causes, though the sanative or healing process might be accomplished by the same means. I do not recollect that modern surgery affords us this disease, under its present Grecian dress, " fungus αἱματώδης ;" probably, a close application to the perusal of the late Nosological authors, might tend to illuminate the mind, now obscured by *ignorance;* the disease, I presume, is somewhat similar to *eccymosis* or *ecchymoma,* produced (not like cancer, from immoderate pressure, or contusions; there is a consequent rupture of one or more of the small blood vessels, which, instead of being diffused, and taken up by the absorbents, the quantity being too large for absorption, is confined ; coagulation takes place, and should there be a " crack" or small fissure externally, a part of the coagulated blood will make its appearance, and thus assumes the form of *fungus* " αἱματώδης," conformably to the term made use of. But, after the present imperfect investigation, what affinity, or analogy, is there between *cancer* and this *coagulated effusion of blood?* As the mode of treatment was something similar to what has been recommended, and used in cancerous complaints, it does not imply, that the *fungus* should partake of the same nature; by no means, it is well known, that *arsenic,* in various forms, has been, and still is used in a variety of diseases, diametrically opposite to *cancer,* as the ague, hooping-cough, cum multis aliis.

Whatever authority or sanction the name of Hey might carry with it, I have seen repeated instances, under my own observation, of the disease being effectually cured without having recourse to *arsenic*; yet, as it is an efficacious remedy, I shall not further comment upon its application; tion; the following, which is one case adduced out of many, will be sufficient to corroborate my assertion.

About two months since, a man of the name of *Lees*, a horse-dealer, applied to me, respecting a violent contusion received upon the *patella*, occasioned by a fall from his horse, on his return from some fair. On examination, I found the knee considerably tumefied and contused; upon which, leeches were applied. The next day found the general swelling much abated, and the patella was surrounded with a large *tumor*, to which was applied a strong solution of *muriated ammonia* in vinegar, which was not productive of much benefit. The distended tumor broke, and a portion of the coagulated blood presented itself to view; and ever attentive to the operations of nature, being frequently an unerring guide, as Hippocrates justly observes, "Νȣσων φύσις ἰαῖρος," rendered assistance by enlarging the opening, and extracted the *coagulum*, which weighed about four ounces, a hard compact substance. As to its "organization," I shall wave an opinion. The edges of the wound were retained by adhæsive plasters, and the cure was complete in about ten days.

### *The good Effects of Carbonic Gas in Mortification.*

The beneficial effects produced from the external application of charcoal, the basis of carbon, in a state of combination, generating fixed air, has long excited the attention of practitioners, particularly Mr. Sandford of Worcester, in conjunction with the late Messrs. Russell and Jeffreys, with whom it was customary to apply fermenting mixtures to sphacelated or mortified parts. From the successful practice of these gentlemen, I have been induced to try the same means, and fortunately for my patients, as well as myself, with equal success, which the following case will bear ample testimony.

James Groves, a person employed by the factors in this town and Birmingham, to carry parcels, &c. to and from the above places, met with an accident by falling over the shafts of a waggon, lying in the road, which lacerated his leg in a dreadful manner, particularly the inferior or lower part of the gastrocnemius externus. Many days had elapsed previous to making application to me; and from inattention on his own part, the limb had become perfectly livid with inflammation and swelling; the wound sphacelated, discharging a putrid and highly offensive ichor. From the very advanced age of the man, being in his his 70th year, I was apprehensive either amputation would be requisite, or death the consequence: however, from mature deliberation, it was thought proper to try the effects of carbonic gas, administering inwardly plenty of red port; the wound being well covered with fine powdered charcoal, ordered a cataplasm of strong ale grounds mixed with oatmeal, to be applied, and to be repeated 4tis horis; previous to which, to wash the wound with aq. ammoni; in the course of two days the inflammation and swelling had disappeared, the sloughs began to separate, and healthy granulations made their appearance; but as the offensive fœtor still continued, persevered in the same plan two days longer, and mixed with charcoal fresh yeast; at the expiration of which, the wound assumed a different aspect, the sloughs were completely separated, the putrid discharge corrected, healthy granulations produced, also a bland and well digested pus; merely simple dressings are now required, and the wound, though extensive, is filling up, and no doubt will be cicatrized much sooner than could have been expected in a subject unfavourable from age and infirmities.

In an extensive surgical practice, I have witnessed the good effects of the acetosa hort. in similar cases, but more particularly in scrophula, either the leaves made warm and applied to the ulcer, or in the form of cataplasm, where there has been an evident deficiency of the vital principle. The practice of oxygenating indolent ulcer is of ancient date, but not less deserving our attention; no doubt, the many boasted cures effected by *Empirics*, have been by the liberal use of the *Oxyd of Mercury*.

J. W. WEAVER, Surgeon.

*Walsall.*

